# Supplementary material for: Venomix: a simple bioinformatic pipeline for identifying and characterizing toxin gene candidates from transcriptomic data
Source: PeerJ. 2018 Jul 31;6:e5361. doi: 10.7717/peerj.5361 (PMC6074769; doi:10.7717/peerj.5361)
Supplement: Supplemental Information 3 [file peerj-06-5361-s003.gz › FinalOutput_E-6/Venom_allergen_3_30/finaltree.pdf]

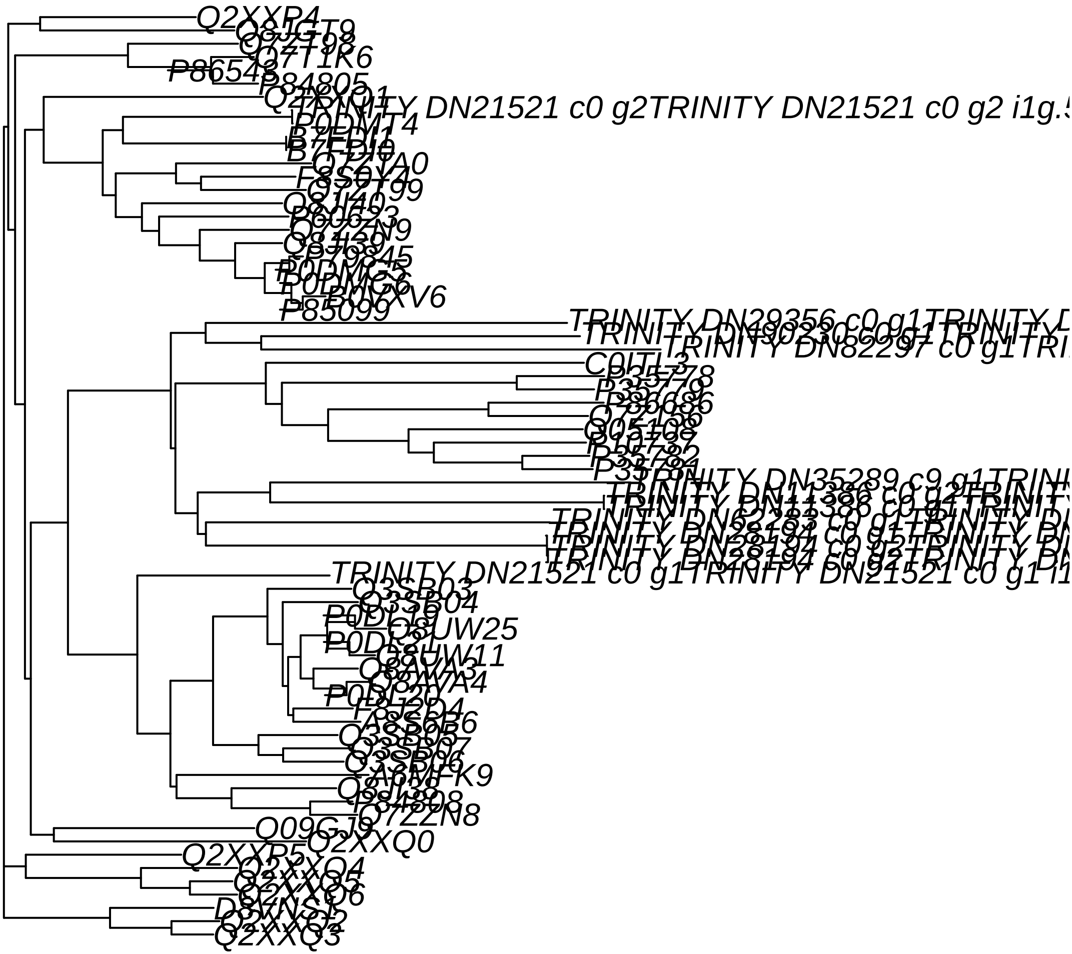
Q2XXP4
Q8JGT9
Q7ZT98
Q7T1K6
P86543
P84805
Q2XXQ1
TRINITY DN21521 c0 g2TRINITY DN21521 c0 g2 i1g.
P0DMT4
B7FDI1
B7FDI0
Q7ZVA0
F8S0Y4
Q92T99
Q8JJ40
P60623
Q7ZZN9
Q8JJ39
P79845
P0DMG5
P0DMG6
P0CXV6
P85099
C0ITL3
P35778
P35786
Q05108
P35782
TRINITY DN21521 c0 g1TRINITY DN21521 c0 g1
Q3SB03
Q3SB04
P0DL19
Q8UW25
Q8UW11
Q8AWA3
Q8AWA4
P0DL20
A8S6B6
Q3SB05
Q3SB07
Q3SB06
A0MFK9
Q7ZZN8
Q09GJ9
Q2XXQ0
Q2XXP5
Q2XXQ4
Q2XXQ5
Q2XXQ6
Q2XXQ2
Q2XXQ3
